# Two Cases of Albumen-Urea in Horses

**Published:** 1892-09

**Authors:** A. W. Clement


### TWO CASES OF ALBUMEN-UREA IN HORSES.

BY A. W. CLEMENT, V. S.

### CASE NO I. POLO PONY.

*History of the Case.*—The animal had been driven, as usual, to a light buggy in the afternoon. He had been in the stable about an hour before anything unusual was noticed. I saw him within two hours after his return to the stable. The owner said that he had not given the horse an unusually hard drive, that he had noticed him to be a little dull, but that he responded to slight urging.

*Clinical History.*—Animal standing in loose box perspiring freely. Respirations forty per minute, nostrils distended, mucous membranes hyperæmic, pupils dilated, locomotion free, no muscular soreness, no evidence of colic, pulse sixty per minute. Considerable arterial tension, temperature 106 degrees, increased murmur, *blowing breathing* in left lung, no rales. There was no perceptible change during the next three days, except that the legs swelled a good deal. Appetite continued good. Quinine had been given during this time. On the third day he was given a large dose of antifebrine, and a cold sponge bath. The temperature dropped two degrees during the next ten hours. On the fourth day I examined his urine, and found it contained a large amount of albumen, should say 30 per cent.

During the next five days the animal continued to improve, and by the thirteenth day after the beginning of the attack the urine was entirely free from albumen, and the pulse, temperature, and respiration normal. The appetite was good, and he seemed lively and well on the road to recovery.

At this time I was called out of town, and was away three nights and two days. About midnight, thirty-four hours after I had left the city, he showed signs of colic. A fellow-practitioner was called in, and when he saw the horse at 4 A. M., he was suffering greatly. During the following day he developed the classical symptoms of enteritis, and died about midnight, twenty-four hours after the beginning of the attack. As my friend was not certain just when I would return, he made an autopsy early in the morning, but for want of time did not examine the organs as closely as he wished and as the clinical symptoms merited. He says that the peritoneal cavity was filled with bloody fluid, that the peritoneum was smooth and of normal color, except in an area about two feet in length, covering the small intestine, where the wall of the intestine showed very dark through the peritoneal covering. This dark area began and ended abruptly. The peritoneal surface over this area was smooth. The mesentery was dark in color and shreddy. There was a verminous aneurism of the mesenteric artery. The mucous membrane of the small intestine was hyperæmic and covered with catarrhal exudate. Other organs, so far as noticed, normal.

### CONCLUSION.

Unfortunately the attending veterinarian did not know the history of the case and did not examine the urine. It is impossible to say whether the last attack had anything to do with the primary trouble or not, and of course, in the absence of very thorough post-mortem investigation, it would be impossible to say whether the case were one, in the beginning, of acute primary nephritis, or whether the albumen urea was a consequence of some infectious trouble. I am inclined to the opinion that the mesenteric aneurism was to blame for the attack which caused the death of the animal.

### CASE NO. 2.

A dark bay gelding, used for farm work, was noticed to be dull and unfit for work one afternoon. He did not eat well that night, and in the morning refused his feed altogether. When I saw him in the afternoon, he presented the following symptoms: Weak, staggering gait, head down, eyes dull and watery, mucous membranes conjested, legs swollen and the skin tense over the swelling. Pulse weak, 50 per minute; respiration normal; lungs normal; temperature 102.3. The following day his urine was caught in a pail hung under his penis by a circingle passed over his loins. During the next twelve hours he passed two quarts of very thick, milky-looking urine. Some of the urine filtered and the clear filtrate examined by the heat and ring test showed a large amount of albumen. Unfortunately the urine was not examined microscopically.

The animal became very weak, but after the end of the first week the albumen began to disappear, and at the end of three weeks he was apparently quite well and able to go to work, but he was allowed to run at pasture, where, I believe, he is at present. It will be interesting to see how his regular work affects him.

